# Carbon-Degrading Enzyme Activities Stimulated by Increased Nutrient Availability in Arctic Tundra Soils

**DOI:** 10.1371/journal.pone.0077212

**Published:** 2013-10-15

**Authors:** Akihiro Koyama, Matthew D. Wallenstein, Rodney T. Simpson, John C. Moore

**Affiliations:** 1 Natural Resource Ecology Laboratory, Colorado State University, Ft. Collins, Colorado, United States of America; 2 Department of Biology, Colorado State University, Ft. Collins, Colorado, United States of America; 3 Department of Ecosystem Science and Sustainability, Colorado State University, Ft. Collins, Colorado, United States of America; Dowling College, United States of America

## Abstract

Climate-induced warming of the Arctic tundra is expected to increase nutrient availability to soil microbes, which in turn may accelerate soil organic matter (SOM) decomposition. We increased nutrient availability via fertilization to investigate the microbial response via soil enzyme activities. Specifically, we measured potential activities of seven enzymes at four temperatures in three soil profiles (organic, organic/mineral interface, and mineral) from untreated native soils and from soils which had been fertilized with nitrogen (N) and phosphorus (P) since 1989 (23 years) and 2006 (six years). Fertilized plots within the 1989 site received annual additions of 10 g N⋅m^-2^⋅year^-1^ and 5 g P⋅m^-2^⋅year^-1^. Within the 2006 site, two fertilizer regimes were established – one in which plots received 5 g N⋅m^-2^⋅year^-1^ and 2.5 g P⋅m^-2^⋅year^-1^ and one in which plots received 10 g N⋅m^-2^⋅year^-1^ and 5 g P⋅m^-2^⋅year^-1^. The fertilization treatments increased activities of enzymes hydrolyzing carbon (C)-rich compounds but decreased phosphatase activities, especially in the organic soils. Activities of two enzymes that degrade N-rich compounds were not affected by the fertilization treatments. The fertilization treatments increased ratios of enzyme activities degrading C-rich compounds to those for N-rich compounds or phosphate, which could lead to changes in SOM chemistry over the long term and to losses of soil C. Accelerated SOM decomposition caused by increased nutrient availability could significantly offset predicted increased C fixation via stimulated net primary productivity in Arctic tundra ecosystems.

## Introduction

The soils of the northern circumpolar permafrost region, which includes Arctic tundra, contain approximately 50% of the global organic carbon, despite only encompassing 16% of the total land surface area [1]. This large pool of Arctic soil organic carbon (SOC) formed as a result of slow soil organic matter (SOM) decomposition relative to the net primary productivity (NPP) within the biome [2]. Factors contributing to the slow decomposition rates include low temperature [3,4], anoxic conditions caused by a persistently high water table due to underlying permafrost [2], and nutrient limitation for soil microbial activity [5]. However, these constraints may lessen due to rapid climate change in the Arctic [6], with uncertain consequences for net C exchange. Climate warming could accelerate decomposition of the massive SOC pool, turning the Arctic tundra biome into a significant CO_2_ source [7], resulting in a positive feedback to global warming [8-10]. 

Accelerated decomposition of SOM and nutrient mineralization due to climate warming can lead to increased nutrient availability [3,4]. Net primary productivity of the Arctic tundra is often limited by nutrients, especially nitrogen (N) [5,11]. Past research has demonstrated that increased nutrient availability leads to shrub domination [5,12] including an increased abundance of *Betula nana* in ecosystems currently dominated by tussock sedges [13-15]. Such change in dominant forms of vegetation can alter biogeochemistry in the Arctic tundra [16]. For instance, shrubs have higher NPP than tussocks [4], and produce woody tissue and litter that decays more slowly than tussock litter, which could produce more resistant SOM [17] and decrease the efficiency of SOM formation [18]. Snow trapped by shrubs increases soil temperature during winter [19,20], yet shading by shrubs decreases soil temperature during summer [21]. 

Increased nutrient availability can accelerate mineralization of SOC, a process which is also thought to be nutrient-limited in the Arctic [5,22]. Mack et al. [5] reported that 18 years of N and phosphorus (P) fertilization in a moist acidic tundra ecosystem significantly reduced SOC relative to non-fertilized controls. This was due to increased decomposition of SOM in the lower organic and in mineral soils, which exceeded increased C sequestration associated with stimulated NPP of shrubs [5]. However, Sistla et al. [23] demonstrated that a two-decade-long summer warming experiment in the same Arctic tundra ecosystem increased the depth of soil biological activity and reorganized the soil food web, but did not significantly alter the total quantity of SOC or soil N. Thus, there is uncertainty in how Arctic tundra will respond to predicted future warming, requiring a more mechanistic understanding of the processes that drive biogeochemical cycling in this system. 

Extracellular enzymes are primarily produced by soil microbes including bacteria, Archaea and fungi, and regulate SOM decomposition by hydrolyzing polymeric compounds [24-28]. Because enzymes are N-rich proteins, their production can also be regulated by N availability. Given the expected increase in nutrient availability associated with warming in the future [3,4] and its potential roles in SOM decomposition [5], it is critical to assess how increased nutrient availability affects the concentration and activity of extracellular enzymes that degrade various substrates in Arctic tundra soils. 

In this study, we examined the effect of long-term field N+P additions on the potential activities of extracellular enzymes in an Arctic tundra ecosystem. We selected total seven extracellular enzymes involved in hydrolysis for C and N products and phosphate (Table 1) in two sites subjected to fertilization since 1989 and 2006 (23 and six years, respectively, as of 2011 when this study was conducted). We hypothesized that increased nutrient availability would increase the abundance of enzymes involved in degrading C-rich substrates, but decrease the abundance of enzymes that degrade N-and P-rich substrates; nutrient-limited soil microbes would reduce their resources to obtain N and P, and reallocate them to obtain C. We also assessed temperature sensitivity of the seven enzymes by conducting enzyme assays at four different temperatures. We hypothesized that increased nutrient availability would alter the temperature sensitivity of enzyme activities (measured as activation energy). 

**Table 1:** List of enzymes assayed in this study.

Enzyme	Abbreviation	Function
*β*-Glucosidase	BG	Releases glucose from cellulose
Cellobiohydrolase	CB	Releases disaccharides from cellulose
Xylosidase	XYL	Degrades hemi-cellulose
*α*-Glucosidase	AG	Releases glucose from soluble saccharides
N-acetyl-glucosaminidase	NAG	Degrades chitin
Leucine-amino-peptidase	LAP	Degrades protein into amino acids
Phosphatase	PHOS	Releases phosphate ions from phosphate group

## Materials and Methods

### Study site and sample collection

Soils were collected from the Arctic Long-Term Ecological Research (LTER) site at Toolik Lake, Alaska, USA (68°38’N, 149°38’W) in late July 2011. The mean annual temperature and precipitation are -7 °C and 400 mm, respectively. Approximately half of the annual precipitation falls as snow. The growing season is limited to between 50 and 70 days in July and August with a mean temperature of approximately 10 °C. The area is dominated by moist acidic tussock tundra with vegetation consisting of graminoids (*Eriophorum vaginatum* and *Carex microchaeta*), deciduous shrubs (*Betula nana*), evergreens (*Ledum palustre* and *Vaccinium vitis-idaea*), and mosses (*Sphagnum* spp., *Hylocomium splendens*, and *Aulacomnium* spp.) [12,29-31]. We sampled from two experimental sites subjected to fertilization treatments since 1989 and 2006. The two different sites were located in moist acidic tussock tundra, 175 m apart from each other. At the 1989 site, we sampled from four plots with a high fertilization treatment (10 g N⋅m^-2^⋅year^-1^ as NH_4_NO_3_ and 5 g P⋅m^-2^⋅year^-1^ as P_2_O_5_) and four no-fertilization controls. At the 2006 site, we sampled from three plots with a high fertilization treatment, three with a low fertilization treatment (5 g N⋅m^-2^⋅year^-1^ and 2.5 g P⋅m^-2^⋅year^-1^ in the same forms as the high fertilization treatment), and three no-fertilization controls. Each soil sample were separated into three soil types, organic, organic/mineral interface and mineral soils based on organic matter content and degree of decomposition. Depths of the organic soils varied from 6 to 12 cm, and the interface from 4 to 15 cm. We collected the mineral soils from the top 5 cm of the profile beneath the interface soils. Samples were transported in a cooler on ice to Natural Resource Ecology Laboratory, Colorado State University, Colorado, USA, and stored at 4 °C until analyses were conducted. All the enzyme assays described below were conducted within one month since the collection of the soil samples. We conducted this study under a permit that the Arctic LTER obtained from the United States Bureau of Land Management which owns the land including our sampling locations. We declare that this study did not involve any endangered or protected species.

### Soil property analysis

We analyzed soil water content, SOC and total N contents for all samples. Soil water contents were determined by drying soil samples at 105 °C for 48 hours. To measure SOC and total N contents, samples were first dried out at 60 °C, and ground finely using a Brinkmann Retsch mill (Haan, Germany). Organic C and total N contents of the ground samples were quantified using a LECO TruSpec® (Leco Corporation, St. Joseph, Michigan, USA). 

### Enzyme assays

We quantified potential activities of seven hydrolytic enzymes (Table 1) for each sample using fluorometric techniques [32] modified following Steinweg et al. [33]. We measured the activities of four enzymes hydrolyzing C-rich substrates (BG, CB, XYL and AG), two for N-rich substrates (NAG and LAP) and one for a P-rich substrate (PHOS). Subsamples (1 g for organic and interface soils, and 2.75 g for mineral soils) were homogenized with 91 mL of 50 mM sodium acetate (pH 4.5) using a blender (Waring, New Hartford, CT, USA). Plates with 96 deep-wells were used for the enzyme assay as well as reference standards, in which samples were arranged in columns and different enzymes and standards in rows. Aliquots (800 µL) of each homogenized sample were pipetted into seven wells in one of the 12 columns of a plate using an 8-channel pipetter. When the plate was filled with homogenized samples (up to 12 samples for a plate), 200 µL of each substrate dissolved in DI water was added to each aliquot of sample. Each of the seven substrates (Table 1) was pipetted into wells in a given row (up to 12 wells). The concentration of each substrate was determined based on tests prior to the experiment. We employed 600 µmol·L^-1^ of CB and PHOS for the organic and interface soils, and 200 µmol·L^-1^ for the rest of the substrates so that 200 µL of substrate would not be completely degraded by enzymes during an incubation period [34]. After a lid was firmly placed on the plate after substrate addition, the plate was inverted several times to mix soil samples and substrates well, and immediately placed in an incubator. Reference standards were prepared in a similar manner as the soil samples described above using the same apparatus. In the standard plates, we added fluorescent standards, instead of the substrates, in seven concentrations ranging from zero to up to 600 µM. We used two types of fluorescent standards, 7-amino-4-methylcoumarin (MUC) and 4-methylumbelliferone (MUB). MUC standards were used for LAP, and MUB the others (Table 1). 

We used four different incubation temperatures (5, 15, 25 and 35°C) to assess the temperature sensitivity of potential enzyme activities in soils. For a set of 12 samples, we set up four plates, each of which was incubated at one of the four temperatures. Four additional plates were prepared as reference standards. Of the four standard plates, two were used for MUC and the other two MUB. One set of MUC and MUB standards were incubated at 5°C and the other at 25 °C along with the soils samples. The standard set incubated at 25°C was used to calculate potential enzyme activities at 15, 25 and 35°C [33]. Incubation periods were 23, 6, 3 and 1.5 hours for 5, 15, 25 and 35°C, respectively. 

After incubation, the plates were centrifuged at 350 g for three minutes, the supernatant was removed from each well and pipetted into a corresponding well of a 96-well black plate. Fluorescent activities were immediately measured using an Infinite M500 spectrofluorometer (Tecan, Männedorf, Switzerland) with a set of wavelength at 365 and 450 nm for excitation and emission, respectively. Readings of the fluorescent activities from standards were used to calculate potential enzyme activities for each sample. 

 Activation energy was calculated using potential enzyme activities assayed at four different temperatures for each enzyme and sample. Temperatures and activity values were fit into the Arrhenius equation [34];

$$K=Ae^{\frac{-Ea}{RT}}$$

where *K* is the reaction rate, *A* is the frequency factor, *Ea* is the activation energy, *R* is the universal gas constant, and *T* is the temperature in Kelvin [35]. This equation can be expressed in the following equation;

$$\ln K=(\frac{-Ea}{R})(\frac{1}{T})+\ln A$$

Plotting ln*K* against 1/T, *Ea* can be calculated from the slope of the linear regression [36]. Activation energy does not have energetic meaning, but rather gauges temperature dependence of enzyme activities [37]. Activation energy describes how enzyme activity increases with temperature [38], thus positively correlates with Q_10_ calculated from the same data set. 

### Statistical analyses

We used a mixed-effect ANOVA for statistical analyses with the sites (i.e. the 1989 and 2006 sites) and fertilization levels (i.e. control, low and high) as fixed effects, and blocks as a random effect. All computations were carried out using the package nlme in R [39]. A significance level of *p* ≤ 0.10 was used to assess statistical significance because of the relatively small sample sizes in this study, and all *p*-values are for two-sided confidence intervals.

## Results

**Figure 1:**
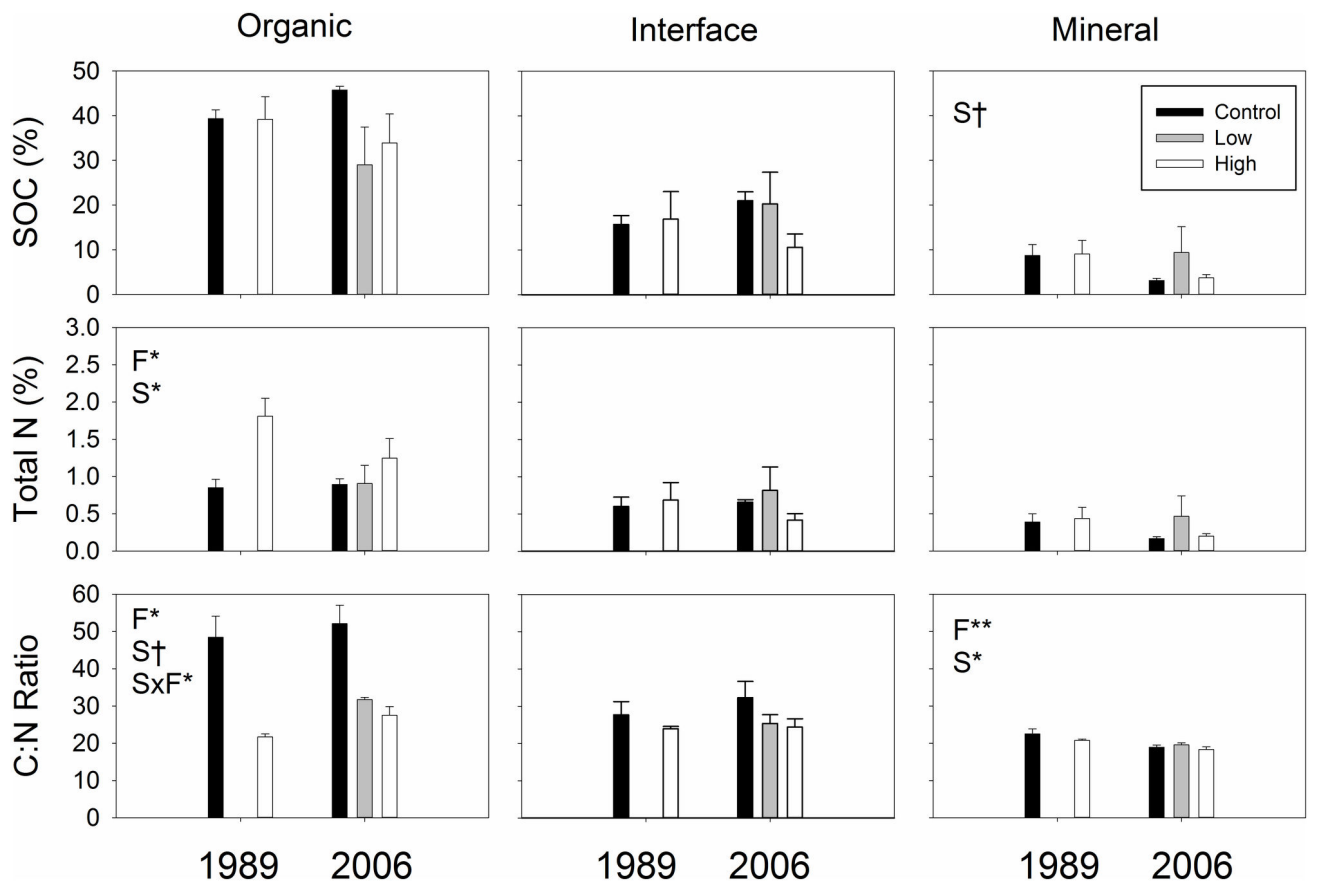
C and total N contents in organic, interface and mineral soil profiles. Statistically significant effects found by mixed-effect ANOVA are shown in panels: F; fertilization treatment (control, low and high fertilizations), S; site (i.e. 1989 and 2006 sites), and F×S; interaction between F and S. Symbols indicate: †; *p* ≤ 0.10, *; *p* ≤ 0.05, **; *p* ≤ 0.01.

 Nutrient addition treatments resulted in increasing the total N content and had no effect on the SOC content in the organic profiles of the soils at the 1989 and 2006 sites (Figure 1). As a result, soil C:N ratios of the organic profiles were significantly reduced with the fertilization treatments (Figure 1). The nutrient addition treatments did not alter either SOC or total N contents in the interface or mineral profiles (Figure 1). C:N ratios of the mineral soil profiles were significantly reduced with the fertilization treatments, though the reduction was small compared to the organic profile (Figure 1). 

**Figure 2:**
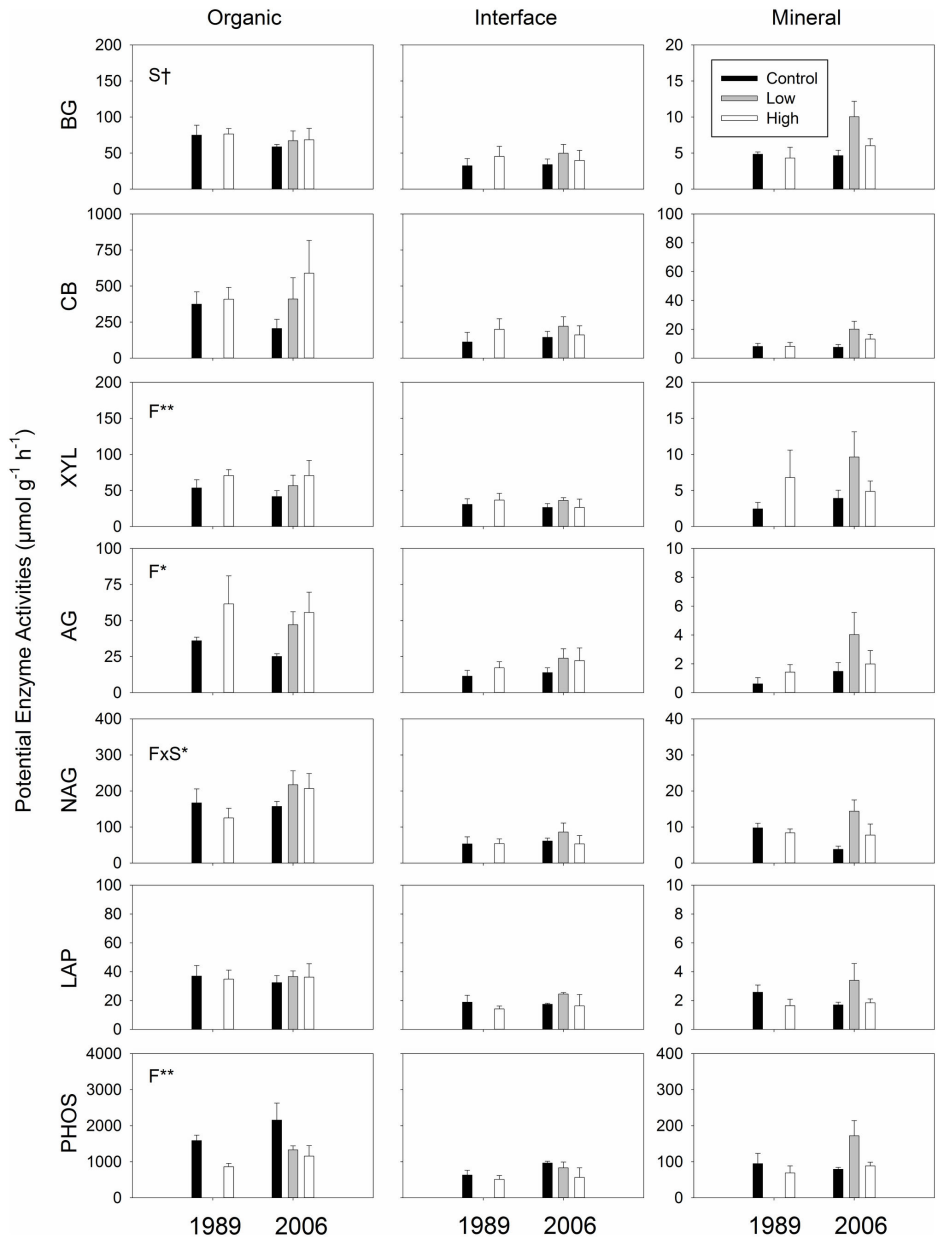
Potential enzyme activities in the three soil profiles, incubated at 15 °C. The scales of y-axes for organic and interface soils are identical. Statistically significant effects found by mixed-effect ANOVA are shown in panels: F; fertilization treatment (control, low and high fertilizations), S; site (i.e., 1989 and 2006 sites), and F×S; interaction between F and S. Symbols indicate: †; *p* ≤ 0.10, *; *p* ≤ 0.05, **; *p* ≤ 0.01.

The effects of increased nutrient availability on potential enzyme activities depended on soil profile and varied by enzyme (Table 2, Figure 2, Appendix S1-4). There was a consistent difference in potential activities of all the enzymes among soil profiles with the activities highest in the organic soils, lowest in the mineral soils and intermediate in the interface soils (Table 2, Figure 2, Appendix S1-4). A significant main effect of fertilization was found on potential activities of CB, AG and PHOS (Table 2, Appendix S4) whereby fertilization increased potential activities of CB and AG but decreased those of PHOS (Figure 2, Appendix S1-3). When potential enzyme activities were analyzed for each soil profile, significant effects were only found in the organic soils (Figure 2, Appendix S1-3). Fertilization stimulated potential enzyme activities of XYL and AG for both 1989 and 2006 sites (Figure 2), whereas fertilization significantly decreased potential enzyme activities of PHOS (Figure 2). Fertilization did not significantly change potential enzyme activities of BG, CB or LAP (Figure 2). 

**Table 2:** Summary of *p*-values resulting from mixed-effect model analyses for potential enzyme activities assayed at 15 °C.

Independent variables	BG	CB	XYL	AG	NAG	LAP	PHOS
Fert	0.342	**0.10**	0.103	**0.006**	0.887	0.316	**0.007**
Profile	**<0.001**	**<0.001**	**<0.001**	**<0.001**	**<0.001**	**<0.001**	**<0.001**
Site	0.736	0.217	0.884	0.287	0.586	0.751	**0.074**
Fert×Profile	0.277	0.613	0.572	0.482	0.898	0.664	0.376
Fert×Site	**0.008**	0.571	0.613	0.920	0.511	0.758	0.839
Profile×Site	0.859	0.341	0.364	0.217	0.209	0.856	0.824
Fert×Profile×Site	0.178	0.211	0.797	0.767	0.326	0.804	0.387

Fert and Profile represent fertilization and soil profile, respectively.

The *p*-values equal to or less than 0.10 are shown bold.

**Figure 3:**
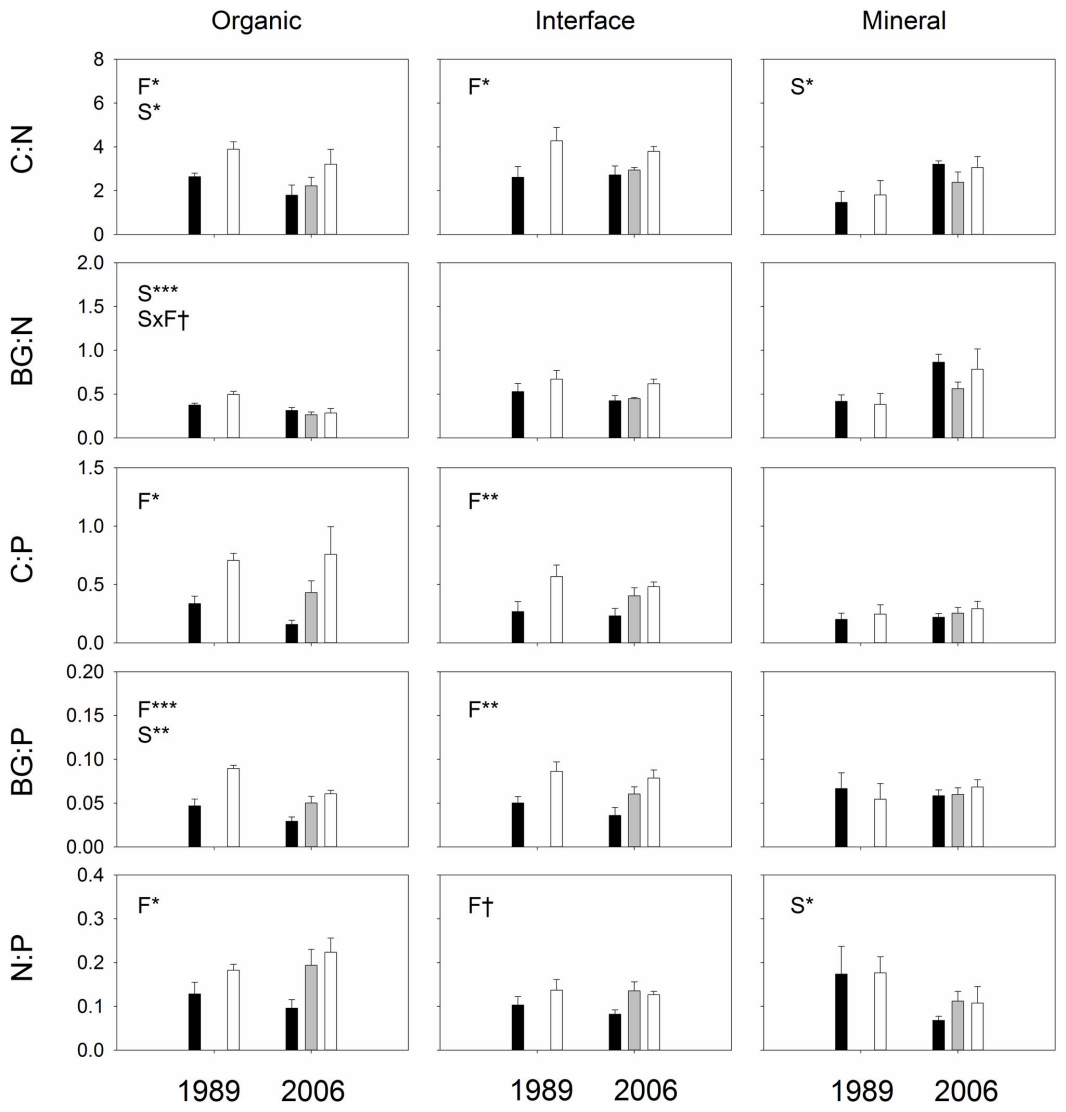
Stoichiometry of potential enzyme activities in the three soil profiles collected assayed at 15 °C. C, N and P represent potential enzyme activities of (BG+CB+XYL+AG), (NAG+LAP) and PHOS, respectively. Statistically significant effects found by mixed-effect ANOVA are shown in panels: F; fertilization treatment (control, low and high fertilizations), S; site (i.e., 1989 and 2006 sites), and F×S; interaction between F and S. Symbols indicate: †; *p* ≤ 0.10, *; *p* ≤ 0.05, **; *p* ≤ 0.01, ***; *p* ≤ 0.001.

**Figure 4:**
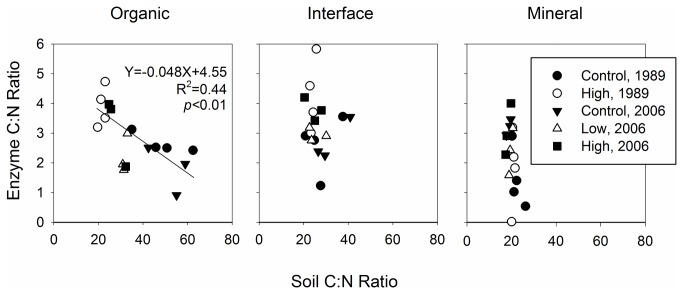
Relationship between soil C:N ratio and corresponding ratio of potential enzyme activities. Enzyme C and N are (BG+CB+XYL+AG) and (NAG+LAP), respectively, assayed at 15 °C.

Fertilization significantly changed the stoichiometry of enzyme, also known as enzyme acquisition ratios (Figure 3, Table 3, Appendix S5-8). Increased nutrient availability significantly stimulated potential activities of C-degrading enzymes relative to N-degrading enzymes as well as phosphatase (Figure 3, Table 3, Appendix S5-8). Such alterations were evident in organic and interface soils, but not in mineral soils (Figure 3, Appendix S6-8). Ratios of BG:N, a commonly used enzyme stoichiometric index [40], was not altered by the fertilization treatments, whereas the BG:PHOS ratio significantly increased (Figure 3). There was a significant correlation between soil C:N ratio and corresponding enzyme ratio in the organic profile (Figure 4). The fertilization treatment reduced soil C:N ratio by increasing soil N content (Figure 1) whereas the treatment increased enzyme C:N ration by increasing C-degrading enzyme activities (Figure 2), resulting in a negative, significant correlation between the two ratios in the organic soils (R^2^=0.44, *p* < 0.01, Figure 4). Such significant correlation was not found in the interface or mineral soils (Figure 4). 

**Table 3:** Summary of *p*-values resulting from mixed-effect model analyses for stoichiometry assayed at 15°C.

Independent variables	C:N	BG:N	C:P	BG:P	N:P
Fert	**<0.001**	0.432	**<0.001**	**<0.001**	**<0.001**
Profile	**0.006**	**0.001**	**<0.001**	0.615	**0.013**
Site	0.897	0.747	0.711	0.119	0.411
Fert×Profile	**0.091**	0.121	**0.012**	**0.008**	0.235
Fert×Site	0.559	0.771	0.378	0.615	0.165
Profile×Site	**0.003**	**<0.001**	0.333	0.218	**0.011**
Fert×Profile×Site	0.810	0.610	0.580	0.513	0.792

C, N and P represent potential enzyme activities of (BG+CB+XYL+AG), (NAG+LAP) and PHOS, respectively.

Fert and Profile represent fertilization and soil profile, respectively.

The *p*-values equal to or less than 0.10 are shown bold.

**Figure 5:**
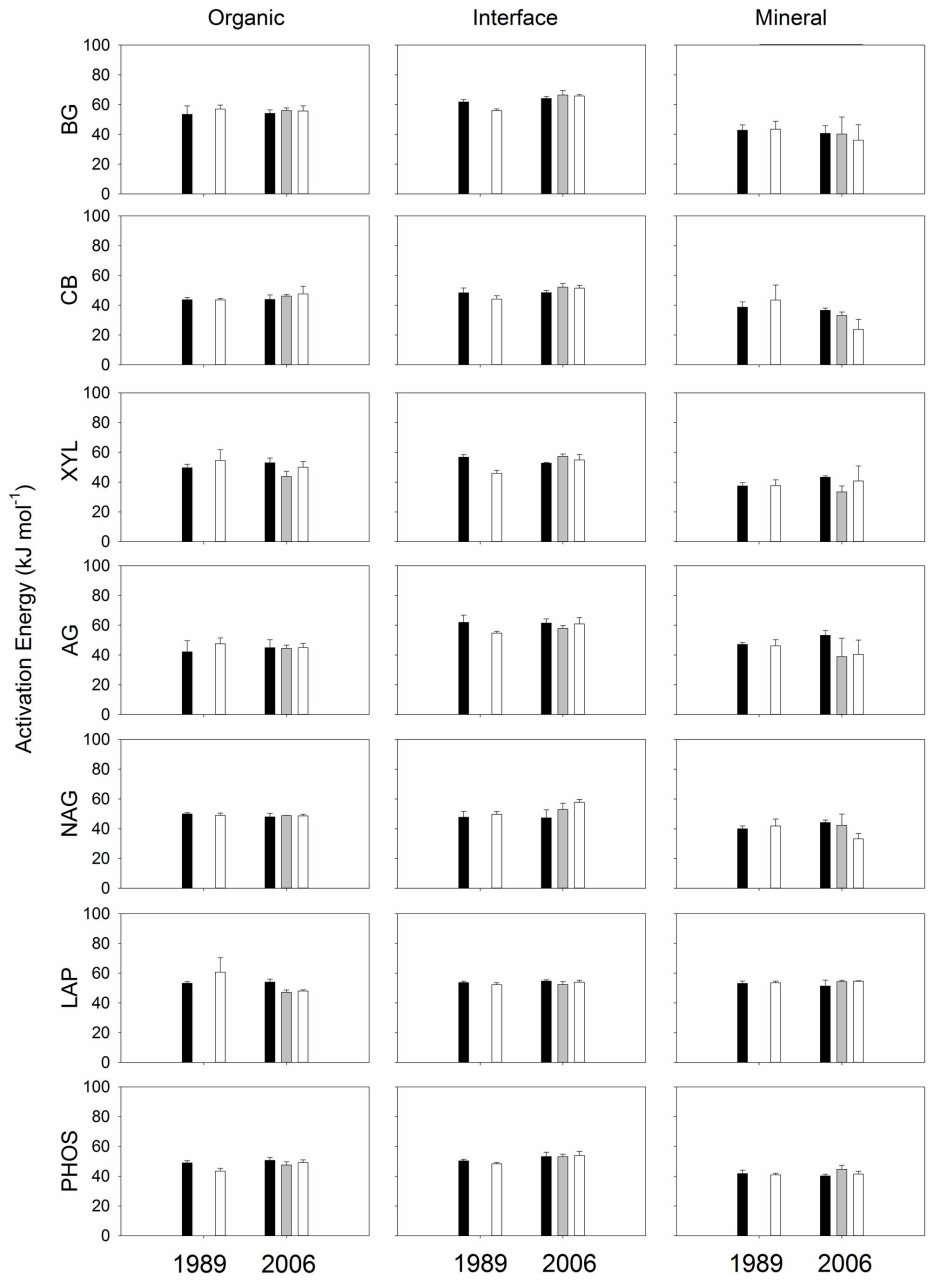
Activation energy of potential enzyme activities. Potential enzyme activities measured at four temperatures (5, 15, 25 and 35 °C) were used to calculate activation energy for the three soil profiles collected from the two sites.

Fertilization did not change activation energy for any of the enzymes assayed in this study (Figure 5, Table 3). There was significant difference in activation energy among the three soil types for all the enzymes except LAP (Table 3), where mineral soils consistently had lower activation energy than organic or interface soils (Figure 5). 

## Discussion

Our study demonstrated that increased nutrient availability in an Arctic tundra ecosystem stimulated the activities of extracellular enzymes that degrade C-rich compounds, which are the proximate drivers of SOC decomposition. Furthermore, nutrient additions altered enzyme acquisition stoichiometry, suggesting that microbes reallocated resources towards obtaining C rather than N or P. This accelerated SOC loss caused by increased nutrient availability, which is expected to happen with climate warming, could significantly offset predicted increased C fixation via stimulated NPP in Arctic tundra ecosystems [41]. 

### Soil properties and potential enzyme activities altered by the fertilization treatments

Our results support our first hypothesis that fertilization would increase potential enzyme activities that degrade C-rich substrates (Figure 2, Appendix S1-3). This result suggests that production of these enzymes by soil microbes is nutrient limited, especially by N [19,42]. During the growing season, net N-immobilization is often observed in Arctic tundra soils [3,19,43-46]. Wallenstein et al. [42] observed that most potential enzyme activities did not change over the course of a growing season in the same Arctic tundra ecosystem, despite increasing temperatures, most likely due to N-limitation for production of enzymes by soil microbes. N-limitation for enzyme production is also supported by Hobbie and Gough [47] who showed that litter decomposition rates in acidic tundra ecosystems were twice as fast as those in non-acidic tundra, most likely due to the higher N availability in the acidic tundra. Increases in C-degrading hydrolytic enzyme activities caused by N addition have been observed in other biomes, including boreal forests [48], temperate deciduous forests [32,49-52], and grasslands [51,53-55]. Thus, our results provide additional support for the general hypothesis that enzyme production is N-limited.

We note that potential enzyme activities reflect overall enzyme concentrations in soils [56], which are determined by the balance between enzyme production and degradation rates. We assume that altered potential enzyme activities by nutrient addition found in this study were caused by microbial responses in enzyme production rates rather than enzyme degradation rates. This assumption is based on our finding that potential activities of LAP, which degrades proteins including enzymes themselves, were not altered by increased nutrient availability (Table 2).

Our results also supported our second hypothesis that increased nutrient availability would decrease phosphatase activities (Figure 2). Decreases in phosphatase activities caused by P fertilization have been commonly observed [57-60] and an inverse relationship between phosphatase activity and environmental P availability is generally accepted [61]. These responses are consistent with a resource economics theory for enzyme production by soil microbes [62]. 

In contrast to C-degrading enzymes and phosphatase, the fertilization treatments did not affect two enzymes that degrade N-rich substrates (i.e., NAG and LAP, Figure 2, Appendix S1-3). Many studies have been conducted to investigate effects of N amendments on these two enzymes across a range of ecosystems, including grasslands [41,54,55,63], temperate forests [49-52] and alpine tundra [64], but there is no consistent trend [61]. One potential reason is that N cycle is more complex compared to those of C or P. For instance, the two N-rich substrates used in this study, chitin (NAG) and protein (LAP), are both C sources as well as N [61]. Thus, even if N-limitation to soil microbes is alleviated, they may still keep producing these two enzymes to acquire C from these substrates. Another potential explanation is that N is still limiting to enzyme production for soil microbes in this system, even after years of chronic N additions.

 Enzyme acquisition ratios, which provide insights into metabolism of soil microbial communities for energy and nutrient acquisitions, were significantly altered by the fertilization treatments (Figure 3, Appendix S6, 7) indicating that soil microbes reallocated their resources to obtain more C. This result emphasizes our finding that enzyme production by soil microbes is nutrient limited. Enzyme expression is determined by microbial cellular metabolism in response to environmental signals, including nutrient availability [40,61]. Thus, shifts in enzyme stoichiometry indicate relative demand of nutrients that soil microbes need for growth and maintenance. One notable finding is that there were significant fertilization effects on enzyme stoichiometry for the interface soils (Figure 3, Appendix S7) even though we did not find any significant fertilization effect when assessing potential activities of individual enzymes (Figure 2, Appendix S2), most likely because of small sample sizes in this study and heterogeneity of the soil samples. 

We found a significant negative correlation between soil C:N ratio and the corresponding ratio of potential enzyme activities in the organic soils (Figure 4) due to contrasting responses of soil and potential enzyme activities to the fertilization treatments. The treatments reduced soil C:N ratio by increasing total N content with SOC content unchanged (Figure 1). The fertilization treatments increased enzyme C:N ratio by increasing activities of enzymes degrading C-rich substrates with no change in N-degrading enzymes (Figure 2). Soil C:N ratios in the controls, approximately 50 in average (Figure 1), are among the highest for organic soils reported in Arctic tundra in the region [65], reflecting high C:N ratios of litter, ranging from 40 to over 100, produced by various plants in the ecosystem [47]. The fertilization treatments reduce such high C:N ratio of organic soils at an early stage of decomposition to ones of highly decomposed soils found in the interface and mineral soils of the controls (Figure 1 and 4) [66]. We did not find such significant, negative correlation in interface or mineral soils (Figure 4) most likely because the C:N ratios of those highly decomposed soils were so low that the fertilization treatments could not lower them further (Figure 1). 

 We note a limitation of this study due to the one time measurement. Potential enzyme activities in Arctic soils can be dynamic over a course of a growing season [42]. Thus, our findings of potential enzyme activities could have been different if measured in other time of the growing season. However, our measurement was conducted around the time when soil temperature peaked, thus SOC decomposition rates were most likely highest [67,68]. 

### Activation Energy

We did not find significant fertilization effects on activation energy in any of the seven enzymes assayed (Figure 5, Table 4). Our finding of no change in activation energy indicates that compositions of isoenzymes were not significantly altered by the fertilization treatments. A pool of enzymes that breaks down a polymer in a natural environment consists of a number of isoforms [69,70]. These isoenzymes can have different activation energy [71], thus a change in the composition of isoenzymes would lead to change in activation energy. Our finding is surprising given that fertilization treatments often change community compositions of soil microbes (e.g. [63,72-76] but see 77,78) which produce different isoenzymes [79,80]. Wallenstein et al. [42] reported temperature sensitivity measured as Q_10_ changed along a growing season for six enzymes in soils collected from the same ecosystem as this study, suggesting compositions of isoenzymes changed over the sampling time. Stone et al. [52] observed no change in temperature sensitivity of potential enzyme activities in temperate forest soils when N was amended. 

**Table 4:** Summary of *p*-values resulting from mixed-effect model analyses for activation energy as independent variable.

Independent variables	BG	CB	XYL	AG	NAG	LAP	PHOS
Fert	0.929	0.632	0.424	0.439	0.562	0.972	0.148
Profile	**<0.001**	**<0.001**	**<0.001**	**<0.001**	**<0.001**	0.634	**<0.001**
Site	0.431	0.704	0.856	0.986	0.642	0.269	0.044
Fert×Profile	0.577	0.750	0.509	0.361	0.208	0.616	0.307
Fert×Site	0.731	0.641	0.813	0.663	0.982	0.490	0.181
Profile×Site	**0.091**	**0.015**	0.425	0.762	0.416	**0.086**	0.366
Fert×Profile×Site	0.490	**0.082**	0.192	0.418	**0.099**	0.129	0.935

Fert and Profile represent fertilization and soil profile, respectively.

The *p*-values equal to or less than 0.10 are shown bold.

Activation energies for most of the enzymes in the mineral soils were lower than those in the organic or interface soils (Figure 5, Table 4). One potential explanation for the difference is compositions of isoenzymes produced in different soil profiles. For instance, organic soils were subject to temperature increase during a growing season, whereas the mineral soils we collected were kept relatively cold in the deeper profiles above permafrost. Such temperature difference in soil profiles where enzymes were produced could contribute to kinetic characteristics of enzymes; the activation energy of psychrophilic enzymes is lower than that of mesophilic enzymes [38]. Another potential explanation is stabilization of enzymes. Enzymes can be stabilized with humic acids [81-83], tannic acids [83,84], and clay minerals [85-87] increasing activation energy compared to free enzymes [88]. Humic and tannic acids in organic and interface soils might contribute to higher activation energy than the mineral soils. Psychrophilic microbes produce more exopolysaccharides than mesophilic microbes [89]. Exopolysaccharides can also affect activation energy as exopolysaccharides stabilize extracellular enzymes and prevent enzyme diffusion [90].

## Conclusions

Our results suggest that nutrient availability limits enzyme production, and thus constrains SOM decomposition in this Arctic tundra ecosystem. Our results indicate that SOM could decompose more rapidly in response to climate change; SOM decomposition stimulated by higher temperature increases nutrient availability, which may act in a synergetic manner. Such accelerated SOM decomposition could stimulate NPP and result in an increase in shrub abundance, which would feedback to alter SOM quantity and quality. A new balance between C sequestration by vegetation and SOC mineralization will determine whether Arctic tundra ecosystems become a C sink, source or vary across space and time. Recently, enzymes have been incorporated as a factor regulating SOM decomposition in several process models to simulate biogeochemical dynamics (e.g. [91,92]). Responses of enzyme activities to increased nutrient availability could provide a mechanistic basis for predicting long-term feedbacks between climate change, microbial activity, and soil C sequestration. 

## Supporting Information

Appendix S1 **Potential enzyme activities in the organic soils assayed at 5, 25 and 35 °C.** Statistically significant effects found by mixed-effect ANOVA are shown in panels: F; fertilization treatment (control, low and high fertilizations), S; site (i.e. long- and short-term fertilization sites), and F×S; interaction between F and S. Symbols indicate: †; *p* ≤ 0.10, *; *p* ≤ 0.05, **; *p* ≤ 0.01.
(TIF)

Appendix S2 **Potential enzyme activities in the *interfacesoils* assayed at 5, 25 and 35 °C.** Statistically significant effects found by mixed-effect ANOVA are shown in panels: S; site (i.e. long- and short-term fertilization sites). Symbols indicate: †; *p* ≤ 0.10.
(TIF)

Appendix S3 **Potential enzyme activities in the *mineralsoils* assayed at 5, 25 and 35 °C.**
(TIF)

Appendix S4 **Summary of *p*-values resulting from mixed-effect model analyses for potential enzyme activities assessed at 5, 25 and 35 °C.** Fert and Profile represent fertilization and soil profile, respectively. *p*-values equal to or less than 0.10 are shown bold.
(PDF)

Appendix S5 **Summary of *p*-values resulting from mixed-effect model analyses for stoichiometry assessed at 5, 25 and 35°C.** Fert and Profile represent fertilization and soil profile, respectively. *p*-values equal to or less than 0.10 are shown bold.
(PDF)

Appendix S6 **Stoichiometry of potential enzyme activities in the *organicsoils* assayed at 5, 25 and 35 °C.**
(TIF)

Appendix S7 **Stoichiometry of potential enzyme activities in the *interfacesoils* assayed at 5, 25 and 35 °C.**
(TIF)

Appendix S8 **Stoichiometry of potential enzyme activities in the *mineralsoils* assayed 5, 25 and 35°C.**
(TIF)
