# Supplementary material for: Carbon-Degrading Enzyme Activities Stimulated by Increased Nutrient Availability in Arctic Tundra Soils
Source: PLoS One. 2013 Oct 15;8(10):e77212. doi: 10.1371/journal.pone.0077212 (PMC3817314; doi:10.1371/journal.pone.0077212)
Supplement: Appendix S4 — Summary of p-values resulting from mixed-effect model analyses for potential enzyme activities assessed at 5, 25 and 35 °C. Fert and Profile represent fertilization and soil profile, respectively. p-values equal to or less than 0.10 are shown bold. (PDF) [file pone.0077212.s004.pdf]

Temperature	Independent variables	BG	CB	XYL	AG	NAG	LAP	PHOS
5ºC	Fert	0.459	**0.056**	0.116	**0.007**	0.927	0.350	**0.008**
	Profile	**<0.001**	**<0.001**	**<0.001**	**<0.001**	**<0.001**	**<0.001**	**<0.001**
	Site	0.504	0.210	0.854	0.202	0.602	0.684	**0.025**
	Fert×Profile	0.740	0.634	0.594	0.484	0.900	0.701	0.380
	Fert×Site	0.894	0.646	0.625	0.921	0.511	0.774	0.840
	Profile×Site	0.306	0.389	0.389	0.218	0.243	0.874	0.826
	Fert×Profile×Site	0.652	0.184	0.809	0.769	0.295	0.828	0.391
25ºC	Fert	0.317	**0.082**	0.190	**0.042**	0.932	0.350	**0.006**
	Profile	**<0.001**	**<0.001**	**<0.001**	**<0.001**	**<0.001**	**<0.001**	**<0.001**
	Site	0.834	0.370	0.900	0.178	0.900	0.655	**0.023**
	Fert×Profile	0.811	0.568	0.586	0.766	0.966	0.674	0.269
	Fert×Site	0.731	0.572	0.802	0.501	0.683	0.894	0.781
	Profile×Site	0.256	0.681	0.947	0.417	**0.021**	0.724	0.999
	Fert×Profile×Site	0.875	0.245	0.693	0.947	0.581	0.624	0.335
35ºC	Fert	0.766	0.104	0.217	**0.068**	0.839	0.268	**0.010**
	Profile	**<0.001**	**<0.001**	**<0.001**	**<0.001**	**<0.001**	**<0.001**	**<0.001**
	Site	0.754	0.223	0.802	0.217	0.563	0.927	**0.010**
	Fert×Profile	0.924	0.602	0.573	0.382	0.889	0.717	0.221
	Fert×Site	0.887	0.643	0.959	0.773	0.993	0.839	0.940
	Profile×Site	0.380	0.623	0.466	0.823	0.162	0.676	0.887
	Fert×Profile×Site	0.970	0.483	0.960	0.895	0.784	0.398	0.550
