# Supplementary material for: Carbon-Degrading Enzyme Activities Stimulated by Increased Nutrient Availability in Arctic Tundra Soils
Source: PLoS One. 2013 Oct 15;8(10):e77212. doi: 10.1371/journal.pone.0077212 (PMC3817314; doi:10.1371/journal.pone.0077212)
Supplement: Appendix S5 — Summary of p-values resulting from mixed-effect model analyses for stoichiometry assessed at 5, 25 and 35°C. Fert and Profile represent fertilization and soil profile, respectively. p-values equal to or less than 0.10 are shown bold. (PDF) [file pone.0077212.s005.pdf]

Temperature	Independent variables	C:N	BG:N	C:P	BG:P	N:P
5ºC	Fert	**<0.001**	0.447	**<0.001**	**<0.001**	**<0.001**
	Profile	**0.005**	**0.001**	**<0.001**	0.614	**0.014**
	Site	0.775	0.293	0.698	**0.068**	**0.011**
	Fert×Profile	**0.085**	0.137	**0.011**	**0.008**	0.244
	Fert×Site	0.554	0.779	0.378	0.615	0.171
	Profile×Site	**0.002**	**<0.001**	0.332	0.218	**0.012**
	Fert×Profile×Site	0.806	0.630	0.579	0.512	0.797
25ºC	Fert	**<0.001**	0.135	**<0.001**	**<0.001**	**0.002**
	Profile	**0.001**	**<0.001**	**0.009**	**0.004**	**0.084**
	Site	0.691	0.687	0.141	0.236	**0.006**
	Fert×Profile	0.154	0.402	**0.017**	**0.008**	0.208
	Fert×Site	0.723	0.235	0.437	0.518	0.421
	Profile×Site	**0.045**	**0.003**	0.538	0.723	**0.007**
	Fert×Profile×Site	0.592	0.742	0.627	0.627	0.786
35ºC	Fert	**0.001**	0.692	**<0.001**	**<0.001**	**0.005**
	Profile	**0.002**	**<0.001**	**0.002**	**0.020**	**0.100**
	Site	0.922	0.864	0.251	**0.006**	**0.025**
	Fert×Profile	**0.082**	0.816	**0.003**	**0.074**	0.224
	Fert×Site	0.825	0.924	0.631	0.975	0.863
	Profile×Site	**0.092**	**0.050**	0.694	**0.097**	**0.025**
	Fert×Profile×Site	0.952	0.804	0.827	0.408	0.753
